# Current progress of mitochondrial transplantation that promotes neuronal regeneration

**DOI:** 10.1186/s40035-019-0158-8

**Published:** 2019-06-14

**Authors:** Chu-Yuan Chang, Min-Zong Liang, Linyi Chen

**Affiliations:** 1 Institute of Molecular Medicine, National Tsing Hua University, 101, Section 2, Kuang-Fu Road, Hsinchu, 30013 Taiwan; 2 Department of Medical Science, National Tsing Hua University, Hsinchu, 30013 Taiwan

**Keywords:** Mitochondrial dynamics, Mitochondrial therapy, Neurodegenerative diseases, Stroke, Neuronal regeneration

## Abstract

**Background:**

Mitochondria are the major source of intracellular adenosine triphosphate (ATP) and play an essential role in a plethora of physiological functions, including the regulation of metabolism and the maintenance of cellular homeostasis. Mutations of mitochondrial DNA, proteins and impaired mitochondrial function have been implicated in the neurodegenerative diseases, stroke and injury of the central nervous system (CNS). The dynamic feature of mitochondrial fusion, fission, trafficking and turnover have also been documented in these diseases.

**Perspectives:**

A major bottleneck of traditional approach to correct mitochondria-related disorders is the difficulty of drugs or gene targeting agents to arrive at specific sub-compartments of mitochondria. Moreover, the diverse nature of mitochondrial mutations among patients makes it impossible to develop one drug for one disease. To this end, mitochondrial transplantation presents a new paradigm of therapeutic intervention that benefits neuronal survival and regeneration for neurodegenerative diseases, stroke, and CNS injury. Supplement of healthy mitochondria to damaged neurons has been reported to promote neuronal viability, activity and neurite re-growth. In this review, we provide an overview of the recent advance and development on mitochondrial therapy.

**Conclusion:**

Key parameters for the success of mitochondrial transplantation depend on the source and quality of isolated mitochondria, delivery protocol, and cellular uptake of supplemented mitochondria. To expedite clinical application of the mitochondrial transplantation, current isolation protocol needs optimization to obtain high percentage of functional mitochondria, isolated mitochondria may be packaged by biomaterials for successful delivery to brain allowing for efficient neuronal uptake.

## Background

Mitochondria are double-membraned cytoplasmic organelles that generate the majority of adenosine triphosphate (ATP) via oxidative phosphorylation. In addition to energy production, mitochondria also function in the biosynthesis of fatty acids, cellular calcium buffering, and act as a platform to integrate cell signalling circuitry that modulates cell survival, immune response, and autophagy [1, 2]. It has been hypothesized that mitochondria evolved from engulfed prokaryotic bacteria so that they possess their own circular DNA (mitochondrial DNA, mtDNA) encoding 37 genes and 13 mitochondrial proteins. Together with nuclear encoded mitochondrial proteins, they maintain mitochondrial integrity [2–4]. Research in the past decade has unveiled that mitochondria are dynamic bioenergetic organelles undergoing controlled fusion, fission, transport, and targeted turnover. Mitochondrial population and quality are controlled in part by dynamic morphogenesis. Initiation of mitochondrial fission starts with recruiting cytosolic dynamin-related protein 1 (Drp1) to mitochondrial outer membrane and forming Drp1 oligomers at candidate fission site, which is marked by ER-mitochondria contact region. Drp1 oligomers then constrict mitochondrial membrane upon GTP hydrolysis to divide mitochondria [5–7]. Fusion, on the other hand, is initiated by mitofusin-1 and -2 (Mfn1 and Mfn2), which are anchored to the outer mitochondrial membrane (OMM) and mediate fusion of OMM. Fusion of inner membrane (IMM) depends on inner membrane GTPase optic atrophy protein 1 (OPA1), which is spliced into long isoform, L-OPA1, and short isoform, S-OPA1. L-OPA1 is required for IMM fusion while S-OPA1 is associated with mitochondrial fission [5, 6].

The dynamic feature of mitochondria serves to adjust cellular metabolism according to physiological states [8, 9]. During early development, stochastic mitochondrial segregation leads to genetic drift effect, raising the risk of pathogenic homoplasmy and the subsequent mitochondrial dysfunction. Given the maternal inheritance of mtDNA, accumulated mtDNA mutations are very likely to be transmitted to the offspring during fertilization whilst paternal mtDNA is targeted to be destroyed. Consequently, the highly dynamic nature of mitochondria evolves as a compensation to retain mitochondrial heteroplasmy in cells [10]. Mitochondrial fusion requires the fusion of outer and inner mitochondrial membranes to form tubular or elongated interconnecting mitochondrial networks within cells and allows the communication of mitochondrial materials between organelles. As mutated mtDNA accumulates, mitochondrial fusion buffers defective mtDNA by mixing wild-type and mutant mtDNA to compensate mitochondrial function or undergoing mtDNA recombination to prevent homoplasmic inheritance of mutated mtDNA into daughter cells [10]. Mitochondrial fission, in contrast, has mainly been implicated in mitochondrial replication, transport, turnover, and cell survival. During cell division, mitochondria are replicated and split into daughter cells. As part of mitochondrial quality control machinery, mitochondrial fission antagonizes fusion events and prompts segregation of damaged mitochondria for further destruction via mitophagy. Divided smaller mitochondria facilitate mitochondrial transport through interaction with motor proteins along cytoskeletal networks to meet energy demand at distal region. For example, mitochondrial fission and recruitment are prominent in primary cortical neurons during development and in vicinity of dendritic protrusions of hippocampal neurons to benefit the plasticity of spines and synapses [11, 12]. Drp1-dependent mitochondrial fission has been reported to modulate programmed cell death following the recruitment of pro-apoptotic proteins, such as Bcl-2-associated X protein (Bax) and Bcl-2 antagonist. Findings from our laboratory also reveal enhanced mitochondrial fission in response to injury and during regeneration of hippocampal neurons [13].

Brain is highly energy-demanding, consuming about 20% of body’s energy. Thus, mitochondrial localization within dendrites and axons supply energy as well as to maintain calcium homeostasis [14]. It is thus not surprising to find that mitochondrial distribution and transport are essential for synaptogenesis and dendritic spine formation during development as well as for regulating neuronal activity and behaviour [11, 14]. The dependency of neuronal function and structure on mitochondrial integrity and dynamics is echoed by increasing studies that demonstrate mitochondrial dynamic abnormalities in the well documented neurodegenerative diseases, such as Alzheimer’s disease (AD), Parkinson’s disease (PD), Huntington’s disease (HD), ischemic stroke and traumatic brain injury (TBI) [15–17] . To this end, better understanding the mechanism underlying defective mitochondrial dynamics and function in these diseases would provide insights into the improvement of clinical treatment. In this review, we summarize and discuss recent reports that lead to the emerging mitochondrial therapy.

### Mitochondrial dynamics and diseases

#### Neurodegenerative diseases

Due to the complexity and therapeutic setbacks of current treatment for neurodegenerative diseases, increasing attention points to the mitochondria-related pathogenesis [15, 18]. Reduced utilization of glucose in the brain measured by flurodeoxyglucose positron emission tomography (FDG PET) suggests metabolic defect in AD brain and prompts the exploration of the role of mitochondria in AD pathogenesis [19]. In AD, increased S-nitrosylation at Cys644 and phosphorylation at Ser616 of Drp1 protein enhance the GTPase activity and lead to mitochondrial fragmentation [20, 21]. Inhibition of Drp1 in AD models restores amyloid beta (Aβ)-mediated mitochondrial dysfunction, synapse damage, and cognitive impairment. Increase of mitochondrial fragmentation in AD subjects could also result from up-regulated fission proteins (Drp1, Fis1) and down-regulated fusion proteins (Mfn1, Mfn2, OPA1) that partially contribute to gradual neuronal loss and synapse impairment [22–26]. In addition, the absence of an autophagy/mitophagy regulator PTEN-induced putative kinase protein 1 (PINK1) on OMM within neurofibrillary tangles of AD brain fails to recruit Parkin protein upon membrane depolarization and thus underlies the accumulation of damaged mitochondria in AD patients [27]. Intra-hippocampal injection of *PINK1-*expressing construct to transgenic mice that overexpress human form of mutant amyloid precursor protein effectively alleviates Aβ-mediated mitochondrial dysfunction and rescues the mitophagy defect via recruiting autophagy receptors (nuclear dot protein 52 kDa, optineurin) to damaged mitochondria to activate mitophagy signalling [26, 28]. PD-associated leucine-rich repeat kinase 2 (LRRK2) mutant and HD-associated mutant huntingtin protein (mHtt) were found interacting with Drp1 to enhance mitochondrial fission, accompanied by defective anterograde mitochondrial transport and synapse degeneration [29, 30]. The toxicity of mutant PD-associated proteins, including PINK1, Parkin, LRRK2, protein deglycase DJ-1,vacuolar protein sorting-associated protein 35, and α-synuclein, accounts for mitochondrial fission, impaired mitophagy, and neuronal death in the PD genetic models [15, 31]. Loss of synapses concurred with deficiency of mitochondrial complexes I and IV in PD neurons within substantia nigra (SN) were also observed [32]. For HD patients, mHtt protein directly or indirectly alters mitochondrial morphology, functions, bioenergetics status, and dynamics, mainly in the striatum and cortical cerebrum [33, 34]. In addition to mHtt-Drp1-interaction-mediated mitochondrial fission [35], mHtt interacts with OMM and leads to defect of calcium homeostasis. High sensitivity of mitochondria to calcium-induced permeability transition pore in mHtt-expressing clonal striatal cells (conditionally immortalized cells of striatal origin) and striatal neurons results in increased calcium release in the presence of ROS stress [36–38]. However, the clearance of defective mitochondria via mitophagy is inhibited due to the binding of mHtt aggregates to the adaptor proteins, such as p62 and huntingtin-associated protein-1, during formation and transport of autophagosomes [39–41]. Notably, it was demonstrated in HD mice that decreased activity of mitochondrial complex IV and reduced ATP production in striatal cells precede neuronal death [42]. Inhibiting mitochondrial citric acid cycle by administrating 3-nitropropionic acid in the animal models resembles the pathology and symptomatology in HD [43, 44].

#### Stroke

Mitochondrial fission was regarded as an early pathological event in ischemic stroke mice and accompanied by morphological change of mitochondria, high level of free radicals, and ATP depletion [45]. In the middle cerebral artery occlusion (MCAO) mice model, mitochondrial fission occurred in penumbra region 3 h after reperfusion [45]. Another study showed that oxygen–glucose deprivation (OGD)-induced mitochondrial fission resulted in neuronal cell death and inhibition of *Drp1* by siRNA or pharmacological inhibitors prevented mitochondrial fission, reduced death of cortical neurons and reduced the infarct volume in ischemic stroke mice [46]. PINK1 was reported to prevent subcellular translocation of Drp1 and reversed mitochondrial fission induced by OGD. Knockdown of PINK1 caused an increase in fragmented mitochondria and worsened the collapse of mitochondrial membrane potential [47]. The MCAO mice and hypoxic/ischemic condition in hippocampal neurons suppressed the expression of Mfn2. Overexpression of Mfn2 increased the ratio of Bcl-2/Bax and reduced the cleaved caspase 3 and cytochrome c release after hypoxia [48]. These studies indicate that the excess of mitochondrial fission induced by stroke leads to mitochondrial damage and cell death. Thus, restoration of the imbalanced mitochondrial dynamics may potentially be a way to attenuate stroke-induced neuronal death.

#### Traumatic brain injury

**Fig. 1:**
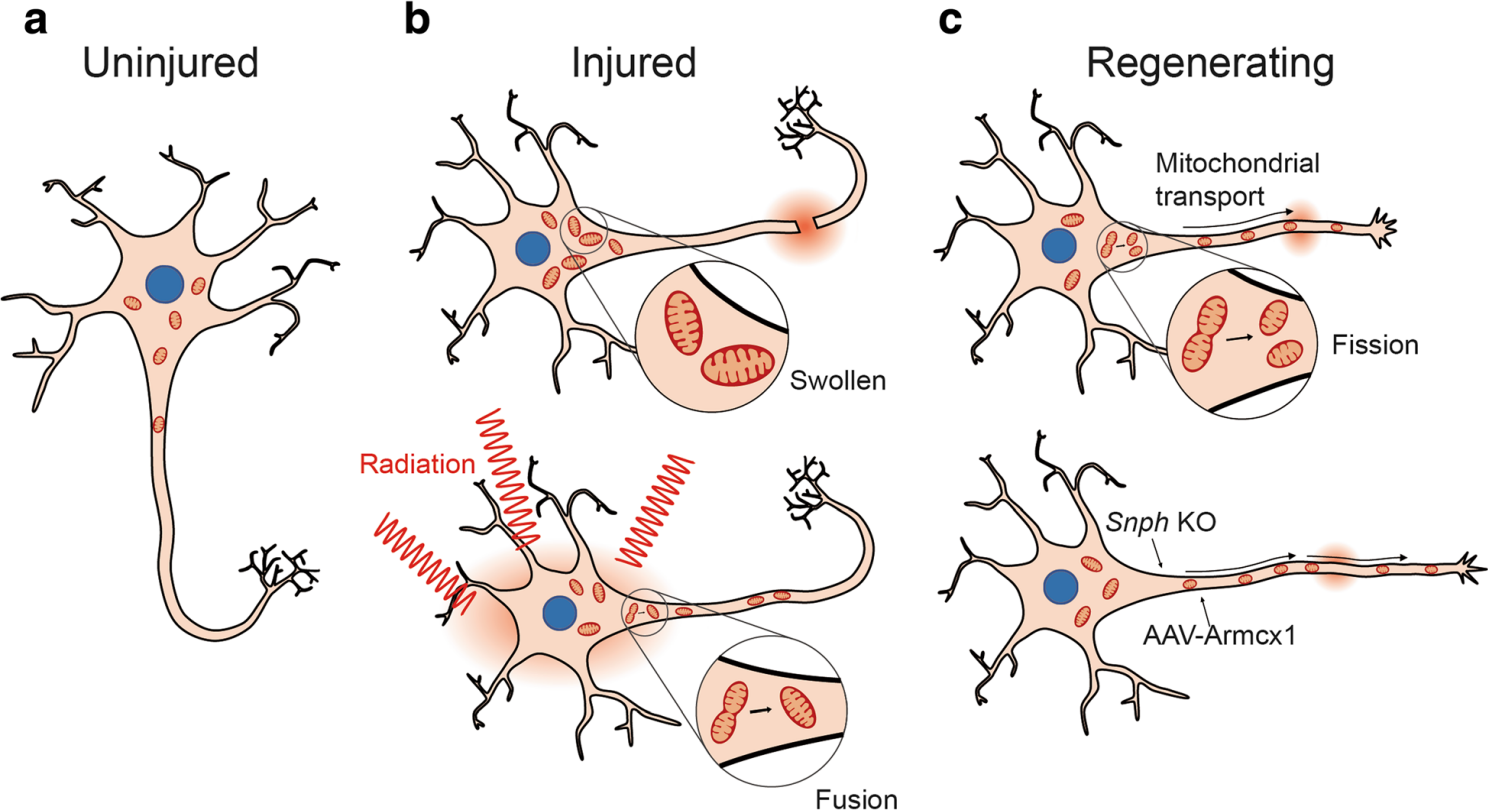
Injury-induced morphogenesis and distribution of mitochondria in neurons. **a** Healthy neurons. **b** (*upper* panel) In response to neuronal injury, the size and number of mitochondria are increased around the axon hillock. (*bottom* panel) Stimuli, such as low-dose ionizing radiation stress, induces mitochondrial fusion [56]. **c** During neuronal regeneration, density of mitochondria and their transport are increased in the regenerating axon. Moreover, knockout of *Snph* or overexpressing Armcx1 have been shown to improve mitochondrial motility and promote axonal regeneration [59, 60]

Studies dated back in 1960s revealed increased number of mitochondria following neuro-axotomy of motor neurons [49, 50]. Mitochondrial swelling were observed in isolated sensory ganglions from limb-amputated newt [51] and in dorsal root ganglions after sciatic nerve crush in rat [52]. Dimova et al. performed axonal section on rat hypoglossal neurons and noted the increased clustering of hypertrophic mitochondria around axon hillock along with strong respiration activity (Fig. 1a and b) [53]. Our previous study reported that fragmented mitochondria were increased 24–48 h after injury in primary hippocampal neurons [13]. Another study showed reduced length of mitochondria in hippocampal neurons after TBI in a controlled cortical impact (CCI) mouse model. The aberrant mitochondrial fission was caused by the increase in Drp1 translocation but not total Drp1 level. Excessive Drp1-mediated mitochondrial fission in TBI animals impairs mitochondrial respiration, leads to reactive oxygen species (ROS) overproduction, and neuronal loss [16]. Mitochondrial division inhibitor 1 (Mdivi-1) treatment attenuated the reduction of mitochondrial length and protected new-born neurons in the hippocampus post injury [16]. A recent study reported that Mdivi-1 blocked the induction of mitochondrial fission and mitophagy in a CCI model of moderate TBI [54]. It appears that TBI induces mitochondrial fission and inhibiting fission can reduce the damage caused by TBI. However, another study on TBI model of rats suggests that the change of mitochondrial fission/fusion dynamics depends on injury severity. The expression level of the genes involved in fission and fusion were down-regulated and up-regulated, respectively, following a mild TBI. In contrast, mitochondrial fission was increased following a severe TBI [55]. Due to the complexity of TBI, it remains debatable whether mitochondrial fission enables higher mobility of mitochondria to the injury site for regeneration or is a result of tissue damage. Nevertheless, these two conclusions do not necessarily conflict with each other.

### A new paradigm of therapeutic strategy: mitochondrial therapy

#### Mitochondrial dynamics and neuronal regeneration

As accumulating data demonstrate the interplay between defective mitochondrial biogenesis and diseases, several lines of evidence reveal dynamic morphogenesis during neuronal regeneration. Our laboratory previously reported that increased mitochondrial fusion promoted survival of hippocampal neurons in response to low-dose ionizing radiation (Fig. 1b) [56]. Interestingly, in response to TBI, mitochondrial fission was increased in hippocampal neurons allowing faster mobilization of smaller/fragmented mitochondria to the injury site, likely to facilitate regeneration process [13]. Along this line, live cell imaging of regenerating neurons after laser axotomy of γ-aminobutyric acid motor neurons of *C. elegans* and Mauthner axons of zebra fish suggests that increased number of mitochondria translocated in injured axons and that mitochondrial mobility is positively correlated with axonal regeneration [57, 58]. Furthermore, genetic knockout of *Snph*, a gene encoding mitochondria-anchoring protein syntaphilin, improved mitochondrial motility in axons after in vivo sciatic nerve injury and enhanced axonal regeneration [59]. Similarly, overexpression of mammalian-specific mitochondrial protein Armcx1in adult retinal ganglion cells mobilized mitochondria in axons and promoted neuronal survival as well as axonal re-growth [60].

These studies raise a possibility that higher mitochondria number and motility in injured neurons may provide better regenerative capacity both in the peripheral nervous system and the central nervous system (CNS) (Fig. 1c) [61–63].

#### Mitochondrial therapy

The concept of “mitochondrial medicine”, which refers to medical intervention targeting mitochondria, boots a new line of biomedical endeavor. Mitochondrial therapy aims to restore mitochondrial functions, such as mildly inducing mitochondrial uncoupling, boosting energy production, and antagonizing the release of ROS. New drugs in forms of mitochondrial membrane uncoupling agents (eg. 2,4-dinitrophenel, uncoupling protein-2, uncoupling protein-3), electron transfer chain-boosting substrates (eg. dichloroacetate, thiamine), metabolism modulators (eg. Metforin) and antioxidants (eg. coenzyme Q_10_, MitoQ, RP103) have been developed or pre-clinically tested [2, 64, 65]. By the end of July 2018, there were more than 400 completed or ongoing clinical trials for mitochondria-targeted medical intervention registered at *ClinicalTrials.gov.* However, there is currently no medicine to cure mitochondria-related diseases caused by inefficient energy production, and the loss of normal physiological ROS function. Therefore, a new paradigm of mitochondrial therapy based on organelle delivery strategy was established. Supplement of healthy mitochondria into cells containing damaged mitochondria was beneficial to improve energy generation, reverse excessive ROS production, and restore mitochondrial function. Findings in recent years have demonstrated the promising outcome upon receiving mitochondrial delivery using in vitro and in vivo models (Table 1) and in several completed or on-going clinical trials (Table 2) [2, 66]. In the following section, we will review recent application of mitochondrial delivery techniques in experimental animals modelling human diseases and highlight the therapeutic potential of delivering isolated mitochondria for the management of neurodegenerative diseases, cerebral stroke, and TBI.

**Table 1:** Summary of the development and recent studies of mitochondrial transplantation

Disease or injury state	Source of mitochondria	Recipient	Method of delivery	Mechanism of mitochondria uptake	Outcome	Reference
In vitro						
Ischemia	MMSCs	Cortical neurons & astrocytes	Co-culture	Cytosol transfer	Improved cell viability	Babenko et al., 2015
UV light damage	PC12	PC12	Co-culture	TNTs	mtDNA transfer	Wang and Gerdes, 2015
Ischemia/reperfusion	BM-MSCs	H9c2	Co-culture	TNTs	Reduced apoptosis process	Han et al., 2016
TBI	Cortical neurons	Hippocampal neurons	Add in medium	Not discussed	Enhanced neuroregeneration	Chien et al., 2018
In vivo						
Acute lung injury (ALI)	mBMSCs; hBMSCs	Aveolar epithelia	Intranasal instillation	Cx43-dependent nanotubes and micro-vesicles formation	Increased alveolar [ATP] and abrogated ALI pathologies	Islam et al., 2012
In situ blood-perfused regional ischemia	Autologous rabbit muscle cells	Myocardial cells	Injection of mitochondria-containing respiration buffer	Actin-dependent organelle-to-cell transfer	Decreased myocyte necrosis and enhanced post-ischemic function	Masuzawa et al., 2013
Transient focal cerebral ischemia	Mouse cortical astrocytes	Peri-infarct cortex	Direct injection or autologous secretions	Integrin-mediated astrocyte-to-neuron mitochondrial transfer	Promoted adjacent neuronal survival and plasticity after injury transfer	Hayakawa et al., 2016
Parkinson’s disease	PC12; human osteosarcoma cybrids	PD rats/ brain neurons	Local injection at medial forebrain bundle	Pep-1-mediated cell-penetrating mitochondrial delivery	Improved locomotive activity and attenuated deterioration of dopaminergic neurons	Chang et al., 2016
Acute myocardial infarction	Autologous porcine muscle cells	Myocardial cells	Injection of mitochondria-containing respiration buffer	Not discussed	Enhanced myocardial cell viability following ischemia and reperfusion	Kaza et al., 2017
Parkinson’s disease	HepG2	Multiple tissues	Intravenous injection	Not discussed	Increased ETC activity, decreased ROS formation, apoptosis and necrosis	Shi et al., 2017
Spinal cord injury: L1/L2 contusion	PC12; syngeneic muscle cells	Brain macrophages, endothelium, pericytes, glia	Microinjection at mediolateral grey matter	Zipper-like actin-mediated phagocytosis	Maintenance of acute mitochondrial bioenergetics, enhanced behavioral recovery	Gollihue et al., 2017
Non-alcoholic fatty liver disease	HepG2	Multiple tissues	Intravenous injection	Not discussed	Decreased lipid content and restored cellular redox balance	Fu et al., 2017
Acetaminophen-induced liver injury	HepG2	Multiple tissues	Intravenous injection	Not discussed	Increased hepatocytes energy supply, reduced oxidation stress	Shi et al., 2018

*MMSCs* mesenchymal multipotent stroma cells, *PC12* pheochromocytoma cell line, *TNTs* tunneling nanotubes, *BM-MSCS* bone marrow-derived mesenchymal stem cells, *H9c2* heart myoblast cell line, *TBI* traumatic brain injury

*m/hBMSCs* mouse/human bone-marrow-derived stromal cells, *Cx43* connexin 43, *HepG2* hepatocellular carcinoma cell line, *ETC* electron transfer chain, *ROS* reactive oxygen species

**Table 2:** Registered interventional studies for mitochondrial transplantation on *ClinicalTrials.gov*

Conditions/Diseases	Status	Phase	Intervention	Mitochondria donor	NCT number
Age-related deterioration of oocyte quality	Withdrawn	1&2	Injection of autologous mitochondria to the oocytes	Autologous granulosa cells	NCT01631578
Infertility	Completed	NA	Autologous micro-injection of mitochondria into the oocytes during ICSI	Autologous ovarian stem cells	NCT02586298
Mitochondrial diseases: Pearson Syndrome	Not yet recruiting	Early 1	Mitochondria augmentation therapy: transplantation of autologous stem cell enriched with MNV-BLD^a^	Autologous peripheral hematopoietic stem cells	NCT03384420
Extracorporeal membrane oxygenation complication	Recruiting	NA	Autologous mitochondria injected or infused into the ischemic myocardium	Autologous skeletal muscle cells	NCT02851758

*NA* not applicable, *ICSI* intracytoplasmic sperm injection, ^a^ MNV-BLD refers to blood-derived mitochondria

### Mechanism of mitochondrial uptake by cells

**Fig. 2:**
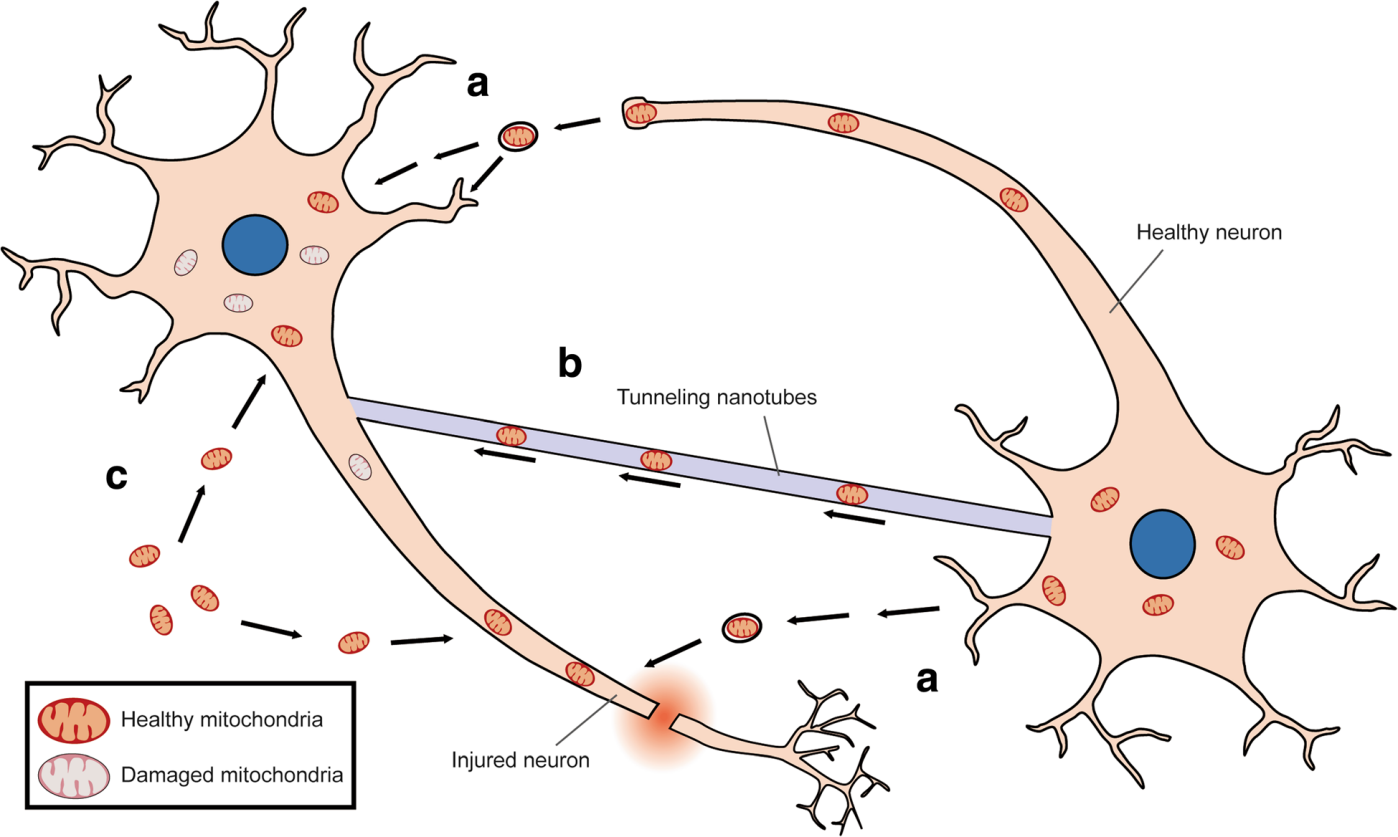
Mechanisms underlying mitochondria internalization. Three uptake routes for mitochondrial therapy: **a** Mitochondria-containing vesicles are released from healthy neurons (or donor cells) and then internalized into injured neurons. **b** Healthy mitochondria are transported via the actin-based tunneling nanotubes between donor cells and injured neurons. **c** Extracellular healthy mitochondria through focal administration are internalized into injured neuron

Mechanisms underlying mitochondrial internalization have been reported (Table 1 and Fig. 2) [67]. Organelle transfer through cell-to-cell fusion or via mitochondria-containing vesicles was observed in bone-marrow-derived stroma cell-to-lung epithelium mitochondrial transfer to mitigate acute lung injury [68]. Tunneling nanotubes (TNTs)-dependent mitochondrial transfer has been well-characterized [68–70]. This actin-based structure was found to mediate mitochondrial exchange between healthy and UV stress-damaged PC12 cells to prevent damaged cells from apoptosis. Nanotube-mediated mitochondrial transfer from co-cultured mesenchymal stem cells to epithelium was reported to rescue cigarette smoke-induced lung damage [71]. Notably, recent study discovered an intriguing mechanism by which stroke-induced activated astrocytes released mitochondria-containing particles and these particles entered damaged neurons through actin-dependent endocytosis to prevent neuronal death [72, 73].

### Mitochondrial delivery for neurodegenerative diseases, cerebral stroke and TBI

As in vivo mitochondrial supplementation in cardiac ischemia models set a milestone for organelle delivery-based therapy, this approach was also applied to neurodegenerative diseases, cerebral stroke, and TBI. Hereinafter, we review the approach of mitochondrial delivery in degenerating, hypoxemic, or injured nervous system.

#### Neurodegenerative diseases

Due to limited understanding of molecular basis underlying AD pathogenesis, available drugs approved by the Food and Drug Administration of the United States for AD, such as acetylcholinesterase inhibitors galantamine, donepezil, and rivastigmine, can simply relieve the symptoms [74, 75]. Since the 1980s, many studies have revealed mitochondrial abnormalities in the AD subjects, including structural change, deficiency of Kreb cycles enzymes, reduced cytochrome oxidase activity, and the disturbance of calcium homeostasis [76–79]. Mitochondrial delivery in AD model was originally conducted in the in vitro cybrid cell system. Cybrids were generated by fusing mtDNA-depleted human neuroblastoma cell line, SH-SY5Y, or teratocarcinoma cells Ntera2/D1 (NT2), with mitochondria from platelets of AD patients [80, 81]. Reduced activity of mitochondrial complex IV, elevated ROS production, higher cytosolic calcium concentration, and defective cytochrome c oxidase, were found in the AD cybrids compared to non-AD control cybrids. Based on these discoveries, mitochondrial cascade hypothesis in the pathogenesis of sporadic AD was then proposed by Khan et al, suggesting that baseline mitochondrial function and durability determine aging-related mitochondrial changes and would progress to AD [82, 83]. Although pre-clinical studies on many anti-oxidants, such as α-tocopherol, for treating AD were found effective in experimental AD animal models, few clinical trials have succeeded. Given the complexity of AD pathophysiology as well as limited efficiency of drug delivery, improved therapeutic strategy of mitochondrial therapy is needed.

Mitochondrial dysfunction aggravates the progression of PD, manifested by increased oxidative stress, dysregulated bioenergetic homeostasis, and reduced viability of affected SN dopaminergic neurons. While mitochondria-targeting antioxidant was considered of great potential for treating PD, existing agents have limited effect on preventing PD from deterioration even if there was promising outcome in animal models and pre-clinical tests [84, 85]. For example, antioxidant drugs, coenzyme Q10 and creatine monohydrate, failed to significantly alleviate the progression in patients with PD in the clinical trials [86, 87]. Therefore, instead of targeting a single specific aspect of mitochondrial function, supplementing healthy mitochondria to damaged regions in PD brain may potentially be an innovative strategy for improving clinical outcome. To this end, several studies set out to examine the efficacy and feasibility of mitochondrial delivery in inhibiting PD progression. Chang et al. demonstrated that cell-penetrating peptide-based mitochondrial delivery in 6-hydroxydopamine (OHDA)-treated PC12 cells rescued mitochondrial respiratory function, improved cell viability, and promoted neurite growth when treated the PC12 cells with nerve growth factor [88]. Xenogeneic/allogeneic injection of mitochondria into medical forebrain bundle (MFB) of 6-OHDA-unilaterally infused PD rats enhanced the survival of dopaminergic neurons as well as effectively sustained mitochondrial functions by restoring the normal level of mitochondrial complex I-IV and relieving mitochondrial oxidative stress in vivo. Upon receiving supplemented mitochondria, protein levels involved in mitochondrial fusion (Mfn2, OPA1), fission (Drp1), and deterioration (Parkin) in dopaminergic neurons within SN were restored. In addition, mitochondrial transplantation in MFB improved locomotive activity of 6-OHDA-induced PD rat. In the other study conducted by Shi et al., MPP (1-methyl-4-phenyl-pyridinium)-treated SH-SY5Y cells incubated with intact isolated mitochondria improved cell viability in a dose-dependent manner [89]. ATP production, mitochondrial complex I activity and cell survival were rescued after mitochondrial supplementation while the level of ROS significantly lowered, compared to MPP^+^ control cells. The initial report by Shi et al. showed that systemic intravenous mitochondrial administration to respiratory chain inhibitor MPTP (1-methyl-4-phenyl-1,2,3,6-tetrahydropyridine)-induced PD mouse model prevented PD progression [89]. In vivo distribution of intravenously-injected mitochondria was found in multiple organs, including brain, 2 h after intravenous injection. As a result, striatal mitochondria in MPTP-induced PD mice showed increased ATP content, restored mitochondrial complex I activity, and decreased ROS production with improved locomotor activity.

#### Stroke

Current intervention for stroke is limited owing to narrow therapeutic time window after the occurrence of ischemic stroke. Ischemia-induced OGD in affected regions leads to low ATP production, excessive ROS release from mitochondria, ionic disequilibrium across mitochondrial membranes, and eventually programmed cell death [17, 90]. As accumulating evidence links mitochondrial deficit to brain impairment following ischemic stroke, therapeutic regimen was developed aiming to restore mitochondrial physiology. In light of new concept of intercellular organelle-transfer, Hayakswa et al. demonstrated that CD38 signalling mediated release of functional mitochondria from activated astrocyte. These mitochondria then entered damaged cortical neurons, restored ATP level and neuronal viability after OGD injury. Treatment with extracellular mitochondria-containing particles, released from cultured astrocytes in a mouse model of focal cerebral ischaemia, provided neuroprotection. In vitro astrocyte-to-neuron mitochondrial delivery and in vivo astrocyte-derived mitochondrial transfer promoted neuronal survival, plasticity, as well as improved behavior outcome [72]. Besides, it has been reported that mitochondria are transferred from mesenchymal multipotent stromal cells to co-cultured neurons. Intravenous administration of mesenchymal multipotent stromal cells to MCAO rats reduced infarction area and improved post-stroke neurological indexes. Treatment of “primed” stem cells, which had been previously co-cultured with neuron cells, caused a more pronounced beneficial outcome in rats after stroke [73]. Transfer of exogenous mitochondria via local intracerebral or systemic intra-arterial injection reduced brain lesion, cell death, and restored motor function in MCAO rats [91]. In addition, autologous mitochondrial transplantation has been studied in rabbit ischemic heart model. After regional ischemia, autologous skeletal muscle-derived mitochondria were injected into ischemic zone of heart prior to reperfusion. Mitochondrial transplantation significantly reduced myocyte necrosis, infarction volume and improved post-ischemic recovery of cardiac function without eliciting any immune or inflammatory response. Moreover, biochemical markers of myocardial infarction, creatine kinase-muscle/brain isoenzyme and cardiac troponin I, were reduced after mitochondrial transplantation [92]. Follow-up study using porcine cardiac ischemia/reperfusion model showed similar results in that autologous mitochondrial transplantation enhanced post-ischemic myocardial cell viability, reduced infarction size and deceased myocardial injury biomarkers [93]. These successful cases highlight the effective mitochondrial therapy in post-stroke neuroprotection, preserving cell viability and promoting functional recovery.

#### Traumatic brain injury

Traumatic injury in the CNS, including spinal cord injury (SCI) and TBI, has been one of the most pressing medical issues worldwide according to its high incidence and lack of effective treatment strategy. The initial study investigating the feasibility of mitochondrial transplantation in SCI reported that supplementation of a pool of healthy mitochondria into L1/L2 contusion SCI rat model acutely sustained cellular bioenergetics in injured spinal cord and improved locomotor activity, whereas long-term effect on neuroprotection and tissue sparing were not observed [94]. On the other aspect, TBI is highly regarded as a global healthcare issue given that it has been the leading cause of injury death according to Center for Disease Control and Prevention, USA [95]. By the end of April in 2018, approximately 69 million of individuals annually suffer from TBI [96]. Post-traumatic mitochondrial deficit includes alternation of membrane structure and calcium homeostasis, uncoupled electron transfer system, accumulation of ROS and induction of apoptosis [97, 98]. Such structural damage and metabolic/physiological dysfunction of mitochondria dampen neuronal viability and plasticity. Disruption of mitochondrial dynamics has also been implicated in TBI-induced behavior impairment and the loss of cognitive function [16, 99]. Accumulating data suggest that mitochondrial therapy could be beneficial for clinical TBI treatment yet the efficacy of mitochondrial transplantation for treating TBI had not been evaluated. A recent report by our laboratory revealed increased mitochondrial fission hours after injury in hippocampal neurons. While retrograde transportation of mitochondria from injury site to cell body was observed in the injured neurites, mitochondria were transported toward newly formed growth cones in re-growing axons. Supplement of freshly isolated mitochondria derived from rat cortical neurons to injured hippocampal neurons promoted neurite re-growth and restored membrane potential of injured neurons [13]. As these findings point to a pivotal role of mitochondrial function in modulating TBI pathophysiology, mitochondrial transplantation could well be a novel strategy for clinical treatment of TBI.

### Clinical application of mitochondrial transplantation

#### Techniques for mitochondrial delivery

The effectiveness of mitochondrial therapy is expected to be variable among patients due to the heterogeneity of pathogenesis and efficiency of mitochondrial internalization into the affected tissues. Successful uptake of mitochondria by target tissues depends on the amount, quality of mitochondria and proper routes of organelle delivery. Therefore, better understanding of the mechanisms underlying mitochondrial delivery and cellular uptake will facilitate the translation of mitochondrial transplantation in clinic.

A number of in vivo studies documented feasible approaches of mitochondrial transplantation, including microinjection directly to affected sites in SCI, stroke, and PD models [88, 92–94], and intravenous administration in PD and fatty liver models [89, 100]. In PD, to improve functional incorporation of supplemented mitochondria, a novel strategy of peptide-mediated allogenic mitochondrial delivery (PMD) was applied to neurotoxin-induced PD rats. Direct microinjection of Pep-1-modified allogenic mitochondria into MFB promoted cellular uptake of mitochondria compared to the injection of naïve mitochondria or xenogenic PMD. It was clear that PMD successfully rescued impaired mitochondrial respiration, attenuated oxidative damage, sustained neuron survival, and restored locomotor activity of PD rats [88]. Nevertheless, the conjugation ratio of Pep-1 and mitochondria should be optimized to avoid undesired mitochondrial aggregation. Moreover, the conjugation time and human manipulation should be minimized before clinical translation. Another study systemically administered isolated mitochondria via tail vein improved locomotor activity in PD mouse model, albeit differential distribution of injected mitochondria in brain, heart, liver, kidney, and muscle [89]. The feasibility of intravenous mitochondrial delivery was achieved by smaller size of the organelle (~ 1 μm in diameter) compared to that of red blood cells (6~8 μm in diameter) and that supplemented mitochondria are not to be incorporated into red blood cells to interfere oxygen transport.

#### Clinical trials

The burgeoning of mitochondrial therapy opened a new era for reversing mitochondria function in human diseases. Thus far, few registered clinical trials for treating neurodegenerative diseases, stroke, or TBI based on mitochondrial delivery technique have been launched. To date, there is only one completed trial which aimed to treat infertility by autologous mitochondrial injection into oocytes (Table 2, NCT#02586298). Autologous ovarian mitochondria were isolated prior to in vitro intracytoplasmic sperm injection (ICSI). The outcome was determined by on-going rate of pregnancy within 12 weeks after mitochondrial therapy, as the improvement in preimplantation genetic screening and embryo quality were also evaluated. An ongoing trial tries to demonstrate the feasibility of mitochondrial transplantation, using autologous mitochondrial injection (Table 2, NCT#02851758), for rehabilitating myocardial ischemia/reperfusion injury and is currently recruiting participants. Mitochondria will be isolated from autologous skeletal muscle from patients undergoing surgical re-operation or catheterization and directly injected into affected myocardium or proximal aorta, or via intracoronary infusion. The outcome will be measured by the safety and the improvement of ventricular function after therapeutic intervention.

## Conclusions

Previous proposals for treating mitochondrial dysfunction have been targeting specific mitochondrial residents and fusion/fission regulators [64, 65]. The outcome of these approaches has not been satisfactory. The emerging line of approach is to supplement freshly isolated mitochondria (mitochondrial transplantation) to injury sites. Alternatively, in the case of stroke, to activate astrocyte for releasing mitochondria-containing particles for inter-cellular transfer of mitochondria (to neurons). Our previous work showed that supplement of freshly isolated mitochondria promoted neurite re-growth and restored the membrane potential of injured hippocampal neurons [13]. Nonetheless, it is conceivable that clinical translation of mitochondrial delivery on TBI would face great challenge. For instance, checkpoint at the blood brain barrier should be considered to improve the effectiveness and the volume used would also be a limiting factor. The therapeutic outcome of mitochondrial transplantation largely depends upon the isolation protocol, quality of isolated mitochondria, and tissue-specific differential uptake. Biocompatible materials for packaging mitochondria may facilitate the delivery and the subsequent uptake by cells. For clinical application, it is more feasible to isolate mitochondria from peripheral tissues to obtain sufficient amount of allogenic mitochondria for the treatment of CNS diseases. Based on our experience, the percentage of functional mitochondria after isolation and the quality maintenance over time are crucial measurement for the success of promoting neuronal regeneration. While published data showed that peptide-based allogeneic mitochondrial delivery successfully entered target cells and recovered damaged tissues without triggering significant immune response in PD model, the efficacy of PMD in cerebral stroke and TBI patients has yet to be determined [88]. More importantly, regenerative outcome characterized by neurite re-growth, de novo synaptogenesis, and the restoration of neuronal activity should be inclusively evaluated in addition to the maintenance of cell survival. Thus, future efforts on the feasibility and efficacy of allogeneic mitochondrial delivery on treating a wide range of mitochondria-related diseases will expedite the clinical translation.

## Data Availability

Not applicable.

## Abbreviations

6-OHDA: 6-hydroxydopamine
AD: Alzheimer’s disease
ATP: adenosine triphosphate
Aβ: amyloid β peptide
Bax: Bcl-2-associated X protein
CCI: controlled cortical impact
CNS: central nervous system
Drp1: dynamin-related protein 1
Fis1: mitochondrial fusion 1 protein
HD: Huntington’s disease
IMM: inner mitochondrial membrane
LRRK2: leucine-rich repeat kinase 2
MCAO: middle cerebral artery occlusion
Mdivi-1: mitochondrial division inhibitor 1
MFB: medical forebrain bundle
Mfn1: mitofusin-1
Mfn2: mitofusin-2
mHtt: mutant huntingtin protein
MPP: 1-methyl-4-phenyl-pyridinium
MPTP: 1-methyl-4-phenyl-1,2,3,6-tetrahydropyridine
mtDNA: mitochondrial DNA
OGD: oxygen–glucose deprivation
OMM: outer mitochondrial membrane
OPA1: optic atrophy protein 1
PD: Parkinson’s disease
PINK1: PTEN-induced putative kinase protein 1
PMD: peptide-mediated mitochondrial delivery
ROS: reactive oxygen species
SCI: spinal cord injury
SN: substantia nigra
TBI: traumatic brain injury
TNTs: Tunneling nanotubes
